# An enhanced RNA alignment benchmark for sequence alignment programs

**DOI:** 10.1186/1748-7188-1-19

**Published:** 2006-10-24

**Authors:** Andreas Wilm, Indra Mainz, Gerhard Steger

**Affiliations:** 1 Institut für Physikalische Biologie, Heinrich-Heine-Universität Düsseldorf, Universitätsstr. 1, 40225 Düsseldorf, Germany

## Abstract

**Background:**

The performance of alignment programs is traditionally tested on sets of protein sequences, of which a reference alignment is known. Conclusions drawn from such protein benchmarks do not necessarily hold for the RNA alignment problem, as was demonstrated in the first RNA alignment benchmark published so far. For example, the twilight zone – the similarity range where alignment quality drops drastically – starts at 60 % for RNAs in comparison to 20 % for proteins. In this study we enhance the previous benchmark.

**Results:**

The RNA sequence sets in the benchmark database are taken from an increased number of RNA families to avoid unintended impact by using only a few families. The size of sets varies from 2 to 15 sequences to assess the influence of the number of sequences on program performance. Alignment quality is scored by two measures: one takes into account only nucleotide matches, the other measures structural conservation. The performance order of parameters – like nucleotide substitution matrices and gap-costs – as well as of programs is rated by rank tests.

**Conclusion:**

Most sequence alignment programs perform equally well on RNA sequence sets with high sequence identity, that is with an average pairwise sequence identity (APSI) above 75 %. Parameters for gap-open and gap-extension have a large influence on alignment quality lower than APSI ≤ 75 %; optimal parameter combinations are shown for several programs. The use of different 4 × 4 substitution matrices improved program performance only in some cases. The performance of iterative programs drastically increases with increasing sequence numbers and/or decreasing sequence identity, which makes them clearly superior to programs using a purely non-iterative, progressive approach. The best sequence alignment programs produce alignments of high quality down to APSI > 55 %; at lower APSI the use of sequence+structure alignment programs is recommended.

## Background

Correctly aligning RNAs in terms of sequence and structure is a notoriously difficult problem.

Unfortunately, the solution proposed by Sankoff [1] 20 years ago has a complexity of *O*(*n*^3*m*^) in time, and *O*(*n*^2*m*^) in space, for *m *sequences of length *n*. Thus, most structure alignment programs (e.g. DYNALIGN [2], FOLDALIGN [3], PMCOMP [4], or STEMLOC [5]) implement lightweight variants of Sankoff's algorithm, but are still computationally demanding. Consequently, researchers often create an initial sequence alignment that is afterwards corrected manually or by the aid of RNA alignment editors (e. g. CONSTRUCT [6], JPHYDIT [7], RALEE [8], or SARSE [9]) to satisfy known structural constraints. The question which alignment technique and/or program performs best under which conditions has been extensively investigated for proteins. The first exhaustive protein alignment benchmark [10] used the so called BAliBASE (Benchmark Alignment dataBASE) [11]. BAliBASE is widely used and has been updated twice since the original publication (BAliBASE 2 and 3, [12,13]). There are a number of other protein alignment databases for example HOMSTRAD [14], OXBench [15], PREFAB [16], SABmark [17], or SMART [18].

These databases contain only sets of protein sequences and, as a reference, high quality alignments of these sets. As a result, emerging alignment tools are generally not tested on non-coding RNA (ncRNA), despite the availability of rather reliable RNA alignments from databases like 5S Ribosomal RNA Database [19], SRPDB [20], or the tRNA database [21].

The BRAliBase (Benchmark RNA Alignment dataBase) dataset used in the first comprehensive RNA alignment benchmark published so far [22] was constructed using alignments from release 5.0 of the Rfam database [23], a large collection of hand-curated multiple RNA sequence alignments. The dataset consists of two parts: the first, which contains RNA sets of five sequences from Group I introns, 5S rRNA, tRNA and U5 spliceosomal RNA, was used for assessing the quality of sequence alignment programs such as CLUSTALW. The other part, consisting of only pairwise tRNA alignments, was used to test a selection of structural alignment programs such as FOLDALIGN, DYNALIGN and PMCOMP. The single sets have an average pairwise sequence identity (APSI) ranging from 20 to 100 %.

Here we extend the previous reference alignment sets significantly in terms of the number and diversity of alignments and the number of sequences per alignment. We present an updated benchmark on the formerly identified "good aligners" and (fast) sequence alignment programs using new or optimized program versions. The performance of programs is rated by Friedman rank sum and Wilcoxon tests. We restricted our selection of alignment programs to multiple "sequence" alignment programs because – at least for the computing resources available to us – most structural alignment programs are either too time and memory demanding, or they are restricted to pairwise alignment. Next, we demonstrate for several programs that default program parameters are not optimal for RNA alignment, but can easily be optimized. Furthermore, we evaluate the influence of sequence number per alignment on program performance. One major conclusion is that iterative alignment programs clearly outperform progressive alignment programs, particularly when sequence identity is low and more than five sequences are aligned.

## Results and discussion

At first we established an extended RNA alignment database for benchmarking (BRAliBase 2.1) as described in Methods. The datasets are based on (hand-curated) seed alignments of 36 RNA families taken from Rfam version 7.0 [24,23]. Thus, the BRAliBase 2.1 contains in total 18,990 aligned sets of sequences; the individual sets consist of 2, 3, 5, 7, 10, and 15 sequences, respectively (see Table 1), with 20 ≤ APSI ≤ 95 %.

To test the performance of an alignment program or the influence of program parameters on performance, we removed all gaps from the datasets, realigned them by the program to be tested, and scored the new alignments by a modified sum-of-pairs score (SPS') and the structure conservation index (SCI). The SPS' scores the identity between test and reference alignments, whereas the SCI scores consensus secondary structure information; for details see Methods. Both scores were multiplied to yield the final RNA alignment score, termed BRALISCORE. For the ranking of program parameters and options of individual programs, or of different programs we used Friedman rank sum and Wilcoxon signed rank tests; for details see Methods. Different program options or even different programs resulted in only small differences in alignment quality for datasets of APSI above 80 %, which is in accordance with the previous benchmark results [22]. Because the alignment problem seems to be almost trivial at these high identities and in order to reduce the number of alignments that need to be computed, we report all results only on datasets with APSI ≤ 80 %.

**Table 1:** Number of reference alignments and average Structure Conservation Index (SCI) for each alignment of *k *sequences.

	k2	k3	k5	k7	k10	k15	total
no. aln.	8976 (118)	4835	2405 (481)	1426	845	504	18990
∅ SCI	0.95 (1.05)	0.92	0.91 (0.87)	0.90	0.89	0.89	0.93

Values for the previously used data-set1 [22] are given in brackets.

### Optimizing gap costs

With the existence of reference alignments specifically compiled for the purpose of RNA alignment benchmarks, program parameters can be specifically optimized for RNA alignments.

**Figure 1:**
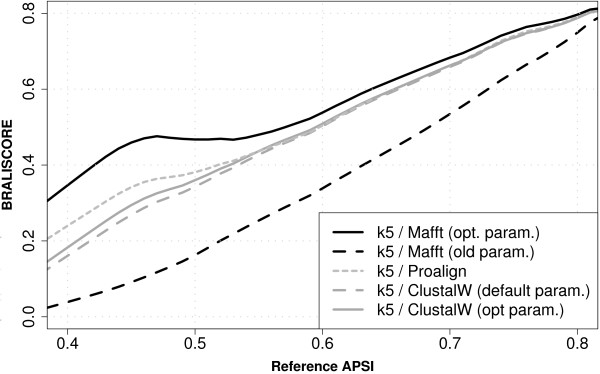
**MAFFT (FFT-NS-2) and ClustalW performance with optimized and old parameters**. PROALIGN (earlier identified to be a good aligner [22]) is included as a reference. Performance is measured as BRALISCORE vs. reference APSI and exemplified for *k *= 5 sequences. MAFFT version 5.667 was used with optimized parameters, which are default in version 5.667, and with (old) parameters of version 4, respectively; CLUSTALW was used either with default parameters or with optimized parameters (see Table 2 and text).

Parameters for MAFFT version 5 [25] have been optimized by K. Katoh using BRAliBase II's data-set1 [22]. The gap-cost values of MAFFT version 4 (gap-open penalty *op *= 0.51 and gap-extension penalty *ep *= 0.041) turned out to be far too low. Applying the improved values (*op *= 1.53 and *ep *= 0.123; these are the default in versions ≥ 5.667) to the new BRAliBase 2.1 datasets results in a dramatic performance gain (exemplified in Figure 1 for alignment sets with five sequences). Similarly, parameters for MUSCLE [16,26] have been optimized by its author.

Motivated by the successful optimizations of MAFFT and MUSCLE parameters, we searched for optimal gap-costs of CLUSTALW [27,28]. We varied gap-open (*go*) and gap-extension (*ge*) penalties from 7.5 to 22.5 and from 3.33 to 9.99, respectively (default values of CLUSTALW for RNA/DNA sequences are *go *= 15.0 and *ge *= 6.66, respectively). Ranks derived by Friedman tests are averaged over all alignment sets, i. e. consisting of 2, 3, 5, 7, 10, and 15 sequences. Table 2 summarizes the results. Alignments created with higher gap-open penalties score significantly better. A combination of *go *= 22.5 and *ge *= 0.83 is optimal for the tested parameter range. It should be noted that this performance gain results mainly from a better SCI, whereas the SPS' remains almost the same.

**Table 2:** Averaged ranks derived from Friedman rank sum tests for ClustalW's gap parameter optimization.

	*ge*							
*go*	0.42	0.83	1.67	3.33	4.99	6.66	8.32	9.99
7.5	56.0	55.0	54.0	53.0	51.2	50.0	47.0	42.8
11.25	47.5	44.0	41.5	37.2	34.5	27.3	28.2	31.5
15.0	20.8	24.0	20.0	14.5	13.5	**15.5**	22.3	29.3
18.75	10.8	8.3	8.2	7.5	11.3	20.8	27.5	35.8
22.5	4.7	**2.8**	3.7	8.8	17.7	27.0	34.5	39.2
26.25	5.8	5.5	8.8	17.5	31.2	36.7	42.3	46.2
30.0	15.2	17.2	22.8	32.8	39.3	45.0	49.0	51.5

Ranks (smaller values mean better performance) for each gap-open (*go*)/gap-extension (*ge*) penalty combination are based on the BRALISCORE averaged over all alignment sets with *k *∈ {2, 3, 5, 7, 10, 15} sequences and APSI ≤ 80 %. CLUSTALW's default and the optimized value combinations are given in bold-face.

Similarly we optimized gap values for the recently published PRANK [29]. Average ranks can be found in Table 3. Default values (*go *= 0.025 and *ge *= 0.5) are too high. Due to time reasons we did not test all parameter combinations; optimal values found so far are 10 times lower than the default values. One should bear in mind that Friedman rank tests do not indicate to which degree a particular program or option works better, but that it consistently performs better. The actual performance gain can be visualized by plotting BRALISCORE vs. reference APSI (see Figure 1). For MAFFT the new options result in an extreme performance gain whereas CLUSTALW gap parameter optimization only yields a modest improvement indicating that CLUSTALW default options are already near optimal. In both cases the influence of optimized parameters has its greatest impact at sequence identities ≤ 55% APSI.

**Table 3:** Averaged ranks derived from Friedman rank sum tests for prank's gap parameter optimization.

	*ge*					
*go*	0.05	0.125	0.1875	0.25	0.375	0.5
0.0025	3.5	2.0	4.8	NA	NA	NA
0.00625	6.8	3.5	3.2	NA	NA	NA
0.00938	8.8	6.5	8.0	NA	NA	NA
0.0125	NA	NA	NA	8.2	11.0	13.5
0.01875	NA	NA	NA	12.8	12.5	15.8
0.025	NA	NA	NA	15.8	17.2	**19.0**
0.03125	NA	NA	NA	20.0	22.0	23.8
0.0375	NA	NA	NA	25.0	27.0	27.8

Ranks (smaller values mean better performance) for each gap-open (*go*)/gap-extension (*ge*) value combination are averaged over all alignment sets with *k *∈ {5, 7, 10, 15} sequences and APSI ≤ 80 %. The default option for PRANK version 1508b is given in bold-face. Values for sets *k*2 and *k*3 are missing because PRANK crashed repeatedly with these sets, but we needed all values to compute the Friedman tests.

### Choice of substitution matrices

Each alignment program has to use a substitution matrix for replacement of characters during the alignment process. Traditionally these matrices differentiate between transitions (purine to purine and pyrimidine to pyrimidine substitutions) and transversions (purine to pyrimidine and vice versa), but more complex matrices have been described in the literature. An example for the latter are the RIBOSUM matrices [30] used by RSEARCH to score alignments of single-stranded regions. To address the question whether incorporating RIBOSUM matrices results in a significant performance change, we used the RIBOSUM 85–60 4 × 4 matrix as substitution matrix for CLUSTALW, ALIGN-M and POA, as these programs allow an easy integration of non-default substitution matrices via command line options. Since gap-costs and substitution matrix values are interdependent we adjusted the original RIBOSUM values to the range of the default values. We applied Wilcoxon tests to test whether using the RIBOSUM matrix (instead of the simpler default matrices) yields a statistical significant performance change. Results are summarized in Table 4. POA and ALIGN-M perform significantly better, only CLUSTALW's performance suffers from RIBOSUM utilization. The reason for CLUSTALW's performance loss is not obvious to us; it might be that CLUSTALW's dynamic variation of gap penalties in a position and residue specific manner [27] works optimally only with CLUSTALW's default matrix. Furthermore, the RIBOSUM 4 × 4 matrix is based on nucleotide substitutions in single-stranded regions whereas we used it as a general substitution matrix. Other matrices, based on base-paired as well as loop regions from a high-quality alignment of ribosomal RNA [31], gave, however, no significantly different results (data not shown).

**Table 4:** Comparison of default vs. RIBOSUM substitution matrix by Wilcoxon tests

Program	k2	k3	k5	k7	k10	k15
ALIGN-M	/	+	+	+	/	/
CLUSTALW	-	-	-	-	-	-
POA	+	+	+	/	/	/

If the use of the RIBOSUM 85–60 matrix resulted in a statistically significant performance increase in comparison to use of the default matrix this is indicated with a "+"; "-" indicates that the default matrix scores significantly better. If no statistical significance was found this is indicated with a "/".

### Effect of sequence number on performance

A major improvement of the BRAliBase 2.1 datasets compared to BRAliBase II is the increased range of sequence numbers per set. This allows, for example, to test the influence of sequence number on performance of alignment programs.

**Figure 2:**
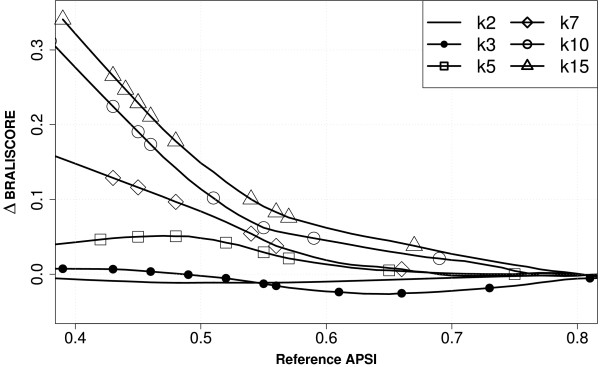
**Performance of Prrn compared to ClustalW in dependence on sequence number per alignment**. The plot shows the difference of the scores of PRRN as a representative of an iterative alignment approach and CLUSTALW (standard options) as a representative of a progressive approach.

It has already been shown that iterative alignment strategies generally perform better than progressive approaches on protein alignments [10]. The same is true for RNA alignments: with increasing number of sequences and decreasing sequence homology iterative programs perform relatively better compared to non-iterative approaches. Figure 2 demonstrates this for PRRN – a representative for an iterative alignment approach – and CLUSTALW as the standard progressive, non-iterative alignment program. The effect is again most notable in the low sequence identity range (APSI < 0.55). In this range, alignment errors occur that can be corrected during the refinement stage of iterative programs. The same can be demonstrated for other iterative vs. non-iterative program combinations like MAFFT or MUSCLE vs. POA or PROALIGN etc. (see supplementary plots on our website [32]).

### Relative performance of RNA sequence alignment programs

To find the sequence alignment program that performs best under all non-trivial situations (e. g. reference APSI ≤ 80 %), we did a comparison of all those programs previously identified [22] to be top ranking. If available we used the newest program versions and optimized parameters. In the comparison we included the RNA version of PROBCONS [33] (PROBCONSRNA; see [34]) whose parameters have been estimated via training on the BRAliBase II datasets. We applied Friedman rank sum tests to each alignment set with a fixed number of sequences. Results are summarized in Table 5. MAFFT version 5 [25] with the option "G-INS-i" ranks first throughout all test-sets. This option is suitable for sequences of similar lengths, recommended for up to 200 sequences, and uses an iterative (COFFEE-like [35]) refinement method incorporating global pairwise alignment information. This option clearly outperforms the default option "FFT-NS-2", which uses only a progressive method for alignment. MUSCLE and PROBCONSRNA rank second and third place.

**Table 5:** Ranks determined by Friedman rank sum tests for all top-ranking programs.

Program/Option	k2	k3	k5	k7	k10	k15
CLUSTALW (default)	8	7	8	8	7	7
CLUSTALW (optimized)	6	6	7	7	6	6
MAFFT (FFT-NS-2)	2	4	4	4	5	5
MAFFT (G-INS-i)	1	1	1	1	1	1
MUSCLE	3	3	3	2	2	2
PCMA	9	10	10	10	10	10
POA	7	8	9	9	9	9
PROALIGN	5	5	6	6	8	8
PROBCONSRNA	4	2	2	3	3	4
PRRN	10	9	5	5	4	3

Programs were ranked according to BRALISCORE averaged over all alignment sets with *k *∈ {2, 3, 5, 7, 10, 15} sequences and APSI ≤ 80 %. MAFFT (G-INS-i) is the top performing program on all test sets. For program versions and options see Methods.

## Conclusion

We have extended the previous "Benchmark RNA Alignment dataBase" BRAliBase II by a factor of 30 in terms of the alignment number and with respect to the range of sequences per alignment. With the new datasets of BRAliBase 2.1 we tested several sequence alignment programs. Obviously it is not possible to test all available programs; here we concentrated on well-known sequence alignment programs and those already identified as good aligners in our first study [22]. Additionally we showed that gap-parameters can be (easily) optimized and tested whether the incorporation of RNA-specific substitution matrices results in a performance change.

From these tests, in comparison with the previous one [22], several conclusions can be drawn:

• While testing the performance of several programs, as for example published in [36], with the *k*5 datasets of BRAliBase II and of BRAliBase 2.1, we found no statistically significant difference of results obtained by the use of these (data not shown); that is, there exists no bias due to the smaller alignment number and the restricted number of RNA families used in BRAliBase II.

• Gap parameter optimization has previously been done only for protein alignment programs. The first BRAliBase benchmark enabled several authors [25] to optimize parameters of their programs for RNA alignments. For example the performance of the previously lowest ranking program MAFFT increased enormously: the new version 5 including optimized parameters [25] is now top ranking.

This result can be generalized: At least the gap costs are critical parameters especially in the low-homology range, but program's default parameters are in most cases not optimal for RNA (e. g. see Tables 2 and 3).

• A further critical parameter set is the nucleotide substitution matrix. We compared the RIBOSUM 85–60 matrix with the default matrix of three programs (see Table 4). The performance of ALIGN-M and POA was either unchanged or improved; however, CLUSTALW performed worse with this RIBOSUM matrix.

• The relative performance of iterative programs (e. g. MAFFT, MUSCLE, PRRN) improves with an increasing number of input sequences and/or decreasing sequence identity. The non-iterative, progressive programs show the opposite trend. With increasing number of sequences and decreasing sequence identity the progressive alignment approach is more likely to introduce errors, which cannot be corrected at a later alignment stage ("once a gap, always a gap" [37]). These errors are corrected by iterative programs during their refinement stage.

• An APSI of 55 % seems to be a critical threshold where the performance boost of (i) iterative programs and of (ii) programs with optimized parameters becomes obvious.

• Given the CPU and memory demand of structure (or sequence+structure) alignment programs, which is mostly above O
 MathType@MTEF@5@5@+=feaafiart1ev1aaatCvAUfKttLearuWrP9MDH5MBPbIqV92AaeXatLxBI9gBamrtHrhAL1wy0L2yHvtyaeHbnfgDOvwBHrxAJfwnaebbnrfifHhDYfgasaacH8akY=wiFfYdH8Gipec8Eeeu0xXdbba9frFj0=OqFfea0dXdd9vqai=hGuQ8kuc9pgc9s8qqaq=dirpe0xb9q8qiLsFr0=vr0=vr0dc8meaabaqaciaacaGaaeqabaWaaeGaeaaakeaaimaacqWFoe=taaa@383D@(*n*^4^) with sequence length *n *and two sequences, the use of BRAliBase 2.1 is too time consuming. Benchmarks with structure alignment programs are possible, however, with a restricted subset of BRAliBase 2.1 or with BRAliBase II (e. g. see [36] and [38]).

Based upon these results we now provide recommendations to users on the current state of the art for aligning homologous sets of RNAs:

1. Align the sequence set with a (fast) program of your choice.

2. Check the sequence identity in the preliminary alignment:

• if APSI ≥ 75 %, the preliminary alignment is already of high quality;

• if 55 % < APSI < 75 %, realign with a good sequence alignment program; at present we recommend MAFFT (G-INS-i) (see Table 5);

• if APSI ≤ 55 %, sequence alignment programs might not be sufficient; structure alignment programs might be of help (e. g. STEMLOC [5], FOLDALIGN [3], etc.), but be aware of memory and CPU usage.

We hope that the BRAliBase 2.1 reference alignments constitute a testing platform for developers, similarly as the BRAliBase II was already used for parameter optimization/training of MAFFT [25], MUSCLE [16,26], PROBCONSRNA [33], STRAL [36], and TLARA [39]. In the future we will try to provide a web interface, to which program authors may upload alignments created with their programs, that are than automatically scored and their performance plotted.

## Methods

The database, which consists of 18,990 sequence set files plus their reference alignments, and scripts used for benchmarking are available [32]. Plots showing BRALISCORE, SCI, and SPS versus APSI for all alignment sets (*k *∈ 2, 3, 5, 7, 10, 15) and for all programs given in Table 5 can also be found there.

### Reference alignments

For the construction of reference alignments we used "seed" alignments from the Rfam database version 7.0 [24,23]. In most cases these alignments are hand-curated and thus of higher quality than Rfam's "full" alignments generated automatically by the INFERNAL RNA profile package [40]. Alignments with less than 50 sequences were discarded to increase the possibility for creation of subalignments (see below). The SCI (see below) for scoring of structural alignment quality is based on a combination of thermodynamic and covariation measures. Thermodynamic structure prediction becomes increasingly inaccurate with increasing sequence length – e. g. due to kinetic effects – but is widely regarded as sufficiently accurate for sequences not exceeding 300 nt in length [41,42]. Thus we excluded alignments with an average sequence length above 300 nt to ensure proper thermodynamic scoring.

To each remaining seed alignment we applied a "naive" combinatorial approach that extracts sub-alignments with *k *∈ {2, 3, 5, 7, 10, 15} sequences for a given average pairwise sequence identity range (APSI; a measure for sequence homology computed with ALISTAT from the squid package [43]). Therefore we computed identities for all sequence pairs from an alignment and selected those pairs possessing the desired APSI ± 10 %. From the remaining list of sequences we randomly picked *k *unique sequences. Additionally we dropped all alignments with an SCI below 0.6 to assure the structural quality of the alignments and to make sure that the SCI can be applied later to score the test alignments. This way we generated overall 18,990 reference alignments with an average SCI of 0.93; the data-set1 used in [22] consists of only 388 alignments with an average SCI of 0.89. For further details see Tables 1 and 6.

**Table 6:** Number of reference alignments for each RNA family

**RNA family**	**k2**	**k3**	**k5**	**k7**	**k10**	**k15**	∑
5S_rRNA	1162	568	288	150	90	50	2308
5_8S_rRNA	76	45	17	5	3	0	146
Cobalamin	188	61	15	4	0	0	268
Entero_5_CRE	48	32	19	10	8	5	122
Entero_CRE	65	38	20	13	8	4	148
Entero_OriR	49	31	17	11	8	4	120
gcvT	167	67	22	12	3	1	272
Hammerhead_1	53	32	9	1	0	0	95
Hammerhead_3	126	99	52	32	17	12	338
HCV_SLIV	98	63	36	26	16	10	249
HCV_SLVII	51	33	19	13	10	7	133
HepC_CRE	45	29	18	11	7	3	113
Histone3	84	59	27	11	7	6	194
HIV_FE	733	408	227	147	98	56	1669
HIV_GSL3	786	464	246	151	95	61	1803
HIV_PBS	188	124	76	55	38	25	506
Intron_gpII	181	82	35	22	11	4	335
IRES_HCV	764	403	205	146	83	47	1648
IRES_Picorna	181	117	75	53	35	25	486
K_chan_RES	124	40	2	0	0	0	166
Lysine	80	48	30	17	7	3	185
Retroviral_psi	89	57	34	24	17	11	232
SECIS	114	67	33	16	11	6	247
sno_14q I_II	44	14	1	0	0	0	59
SRP_bact	114	76	39	19	12	7	267
SRP_euk_arch	122	94	42	21	12	6	297
S_box	91	51	25	12	7	2	188
T-box	18	8	0	0	0	0	26
TAR	286	165	92	62	42	28	675
THI	321	144	69	32	17	5	588
tRNA	2039	1012	461	267	143	100	4022
U1	82	65	26	16	6	0	195
U2	112	83	38	22	14	7	276
U6	30	21	14	7	1	0	73
UnaL2	138	71	43	20	7	0	279
yybP-ykoY	127	64	33	18	12	8	262

∑	8976	4835	2405	1426	845	503	18990

### Scores

Just as in the previous BRAliBase II benchmark [22] we used the SCI [44] to score the structural conservation in alignments. The SCI is defined as the quotient of the consensus minimum free energy plus a covariance-like term (calculated by RNAALIFOLD; see [45]) to the mean minimum free energy of each individual sequence in the alignment. A SCI ≈ 0 indicates that RNAALIFOLD does not find a consensus structure, whereas a set of perfectly conserved structures has SCI = 1; a SCI ≥ 1 indicates a perfectly conserved secondary structure, which is, in addition, supported by compensatory and/or consistent mutations. The SCI can, for example, be computed by means of RNAZ [44]. To speed up the SCI calculation we implemented a program, SCIF, which is based upon RNAZ but computes only the SCI. SCIF was linked against RNAlib version 1.5 [46,47].

In [22] we used the BALISCORE, which computes the fraction of identities between a trusted reference alignment and a test alignment, where identity is defined as the averaged sequence identity over all aligned pairs of sequences. Because the original BALISCORE program has certain limitations and peculiarities, e. g. skips all alignment columns with more than 20 % gaps, we instead used a modified version of COMPALIGN [43] called COMPALIGNP, which also calculates the fractional sequence-identity between a trusted alignment and a test alignment. Curve progressions for scores computed by BALISCORE and COMPALIGNP are only marginally shifted. The COMPALIGNP score is called SPS' throughout the manuscript.

As both scores complement each other and are correlated, we use the product of both throughout this work and term this new score BRALISCORE.

### Statistical methods

**Figure 3:**
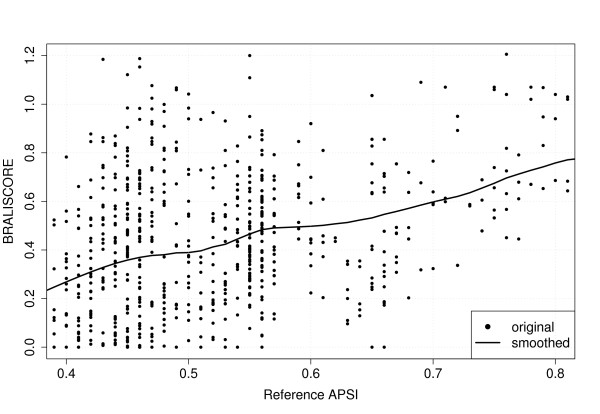
**Lowess smoothing**. The plot shows the scattered data points, each corresponding to one alignment, exemplified by the performance of PROALIGN with *k *= 7 sequences per alignment. The curve is the result of a lowess smoothing with a smoothing factor of 0.3.

The software package R [48] offers numerous methods for statistical and graphical data interpretations. We used R version 2.2.0 to carry out the statistical analyses and visualizations of program performances. For a given APSI value, the scores of the alignments are distributed over a wide range (see for example, in Figure 3 the BRALISCOREs range from 0.0 to 1.2 at APSI = 0.45). Furthermore, the alignments are not evenly spaced on the APSI axis. Thus we used the non-parametric lowess function (locally weighted scatter plot smooth) of R to fit a curve through the data points. The lowess function is a locally weighted linear regression, which also takes into consideration horizontally neighbouring values to smooth a data point. The range in which data points are considered is defined by the smoothing factor. The curve in Figure 3 was computed by a smoothing factor of 0.3, which means that a range of 30 % of all data points surrounding the value to smooth are involved.

For statistical analyses we computed the BRALISCORE for each alignment. To rate the alignment programs or program options, we ranked these scores after averaging over all datasets. Because the score distributions cannot be assumed to be either normal or symmetric, we used as non-parametric tests the Friedman rank sum and the Wilcoxon signed rank test. R's Friedman test was accommodated to calculate the ranking. Afterwards the Wilcoxon test determined which programs or options pairwisely differ significantly. As already shown in [22] programs generally perform equally well above sequence similarity of about 80 %; that is, with such a similarity level the alignment problem becomes almost trivial. To avoid introduction of a bias due to the large number of high-homology alignments with a reference APSI > 80 %, we only used alignments with a reference APSI ≤ 80 % for the statistical analyses.

### Programs and options

The following program versions and options were used:

**ClustalW **: version 1.83[27]

default: -type=dna -align

gap-opt: -type=dna -align -pwgapopen=*GO *-gapopen=*GO  *-pwgapext=*GE *-gapext=*GE *

subst-mat.: -type=dna -align -dnamatrix=*MATRIX *-pwdnamatrix=*MATRIX*

**MAFFT **: version 5.667[25]

default: fftns

default: ginsi

old: fftns --op 0.51 --ep 0.041

old: ginsi --op 0.51 --ep 0.041

**MUSCLE **: version 3.6[16,26]

-seqtype rna

**PCMA **: version 2.0[49]

**POA **: version 2[50]

-do_global -do_progressive *MATRIX*

**prank **: version 270705b – 1508b[29]

-gaprate=*GR *-gapext=*GE*

**ProAlign **: version 0.5a3[51]

java -Xmx256m -bwidth = 400 -jar ProAlign_0.5a3.jar

**ProbConsRNA **: version 1.10[33]

**Prrn **: version 3.0 (package scc)[52]

## Competing interests

The author(s) declare that they have no competing interests.

## Authors' contributions

A.W. developed the BRAliBase 2.1 and performed the benchmark; I.M. developed the ranking tests. All authors participated in writing the manuscript.
